# ThermoAlign: a genome-aware primer design tool for tiled amplicon resequencing

**DOI:** 10.1038/srep44437

**Published:** 2017-03-16

**Authors:** Felix Francis, Michael D. Dumas, Randall J. Wisser

**Affiliations:** 1 Center for Bioinformatics and Computational Biology, University of Delaware, Newark, DE 19714, USA; 2 Department of Plant and Soil Sciences, University of Delaware, Newark, DE 19716-1304, USA

## Abstract

Isolating and sequencing specific regions in a genome is a cornerstone of molecular biology. This has been facilitated by computationally encoding the thermodynamics of DNA hybridization for automated design of hybridization and priming oligonucleotides. However, the repetitive composition of genomes challenges the identification of target-specific oligonucleotides, which limits genetics and genomics research on many species. Here, a tool called ThermoAlign was developed that ensures the design of target-specific primer pairs for DNA amplification. This is achieved by evaluating the thermodynamics of hybridization for full-length oligonucleotide-template alignments — *thermoalignments* — across the genome to identify primers predicted to bind specifically to the target site. For amplification-based resequencing of regions that cannot be amplified by a single primer pair, a directed graph analysis method is used to identify minimum amplicon tiling paths. Laboratory validation by standard and long-range polymerase chain reaction and amplicon resequencing with maize, one of the most repetitive genomes sequenced to date (≈85% repeat content), demonstrated the specificity-by-design functionality of ThermoAlign. ThermoAlign is released under an open source license and bundled in a dependency-free container for wide distribution. It is anticipated that this tool will facilitate multiple applications in genetics and genomics and be useful in the workflow of high-throughput targeted resequencing studies.

Isolating homologous DNA sequence from individuals at specific regions of a genome is fundamental to research and applications in genetics and genomics. Despite the advent of high-throughput sequencing technologies, obtaining a fully contiguous and accurate region-specific consensus sequence for multiple individuals from whole-genome DNA libraries can be cost prohibitive, unnecessary or inefficient. This is particularly challenging for investigations of large genomes and when surveying variation in population studies where hundreds to thousands of individuals are concerned. Consequently, a number of techniques have been devised for targeted enrichment, including microarray-based sequence capture (e.g. refs 1 and 2), molecular inversion probes (e.g. ref. 3)and PCR (e.g. ref. 4), each of which require the design of target-specific hybridization or priming oligonucleotides.

Genomes contain varying degrees of repetitive DNA^5^, and for many species this represents the major fraction of the genome. It has been estimated that >50%^6^ and as much as 69%^7^ of the human genome is repetitive, and over 80% of the genomes for some plant species is repetitive (e.g. refs 8 and 9). This poses a significant challenge to designing oligonucleotides that will hybridize and prime only on-target. Targeted enrichment approaches relying on hybridization-based DNA capture can result in off-target sequences being captured due to nonspecific binding^10^,^11^,^12^. Similarly, primers used for amplification-based approaches may produce off-target products^13^,^14^. This presents the need for “genome-aware” oligonucleotide design tools that leverage reference genome sequence data to maximize the enrichment of on-target sequences.

Although there are several computational tools now available that facilitate genome-aware primer design^15^,^16^,^17^,^18^, obtaining specific amplification of targeted sequences is still a difficult problem, especially for genomes with large amounts of repetitive DNA. A popular tool for choosing primers is Primer-BLAST^19^, which integrates Primer3 for primer design with BLAST and Needleman-Wunsch alignments for evaluating primer specificity. The number of non-complementary bases in sequence alignments is used as the basis for a specificity filter to select candidate primers. However, several factors contribute to the mispriming potential of primers including temperature, reaction chemistry, nucleotide composition and the position and type of mismatching nucleotides^20^, such that the number of mismatches alone is likely to be an insufficient measure of mispriming potential.

Given the availability of empirical data on the thermodynamics of hybridization for complementary^21^ and non-complementary^22^,^23^,^24^,^25^,^26^ base pairings, genome-wide estimates of the thermodynamics of primer-template hybridization can be incorporated into the selection process for oligonucleotide design tools. Indeed, some algorithms have used this approach, including specificity-determining subsequence (SDSS)^13^,^27^,^28^, MFEprimer-2.0^16^ and PRIMEGENSw3^29^. These particular tools differ in terms of the algorithms they use to evaluate priming specificity and to select primer pairs. Nevertheless, for all of these tools, the assessment of mispriming (a critical step for identifying target-specific primers) is restricted because it is initialized by evaluation of specificity at only the 3′-end region of a candidate primer against potential off-target binding sites. Only candidate primers with perfect complementarity at the 3′-end region are further evaluated in terms of binding stability. However, studies have demonstrated that mismatches within the 3′-end region can still lead to PCR amplification^13^,^30^, and Miura *et al*. (2005), who developed SDSS, discussed the need for more sophisticated methods to evaluate off-target hybridization and priming. In this study, such a method was developed which quantitatively evaluates the thermodynamics of hybridization for full-length primer-template binding at the target site relative to the remainder of the genome.

In terms of selecting primers for DNA amplification, most tools assess specificity according to whether primer pairs (not individual primers) are predicted not to produce off-target amplicons within a specified size range (Supplementary Table S1). This criteria can result in the selection of primers that individually produce single-strand synthesis products at multiple off-target sites. For instance, a primer with many perfectly complementary off-target binding sites could be selected by tools that use this approach. In turn, such primers can create byproducts that act as mega-primers in PCR, leading to background amplification^14^. This issue is expected to be exacerbated when working with repetitive genomes. Therefore, evaluating the specificity of individual primers could improve the selection of target-specific primer pairs.

For targeted resequencing, it is still not feasible to enrich and sequence many tens of kilobases or more of contiguous DNA, particularly for large samples of individuals. Some techniques have been developed that might be used for this, but in our attempts neither region-specific enrichment^31^ nor selective whole genome amplification^32^ led to enrichment of targeted regions of the maize genome (Supplementary Methods and Results). Further optimization may enable these techniques to work for our target regions, but our results suggested the repetitive nature of the genome prevented enrichment. Indeed, others have found that hybridization-based enrichment has proven difficult^1^ or unsuccessful^12^ in maize due to its repeat content. In contrast, given primer pairs that specifically prime on-target, DNA amplification is a highly effective method for enrichment. Thus, coupling target-specific primer design for DNA amplification with automated identification of primer pairs that amplify overlapping segments across a target region is an alternative solution to creating libraries for targeted resequencing. Unfortunately, existing tools are not suited for this and output only a subset of the higher scoring primer pairs designed to a region of interest. This leads to parts of the targeted locus not being covered or a situation where users need to manually pick from among numerous possible primer pairs. Therefore, the automated design of groups of primer pairs producing a tiling path of amplicons would be useful for resequencing studies. Some studies have approached the problem of designing amplicon tiling paths by segmentation of the genomic locus and evaluating potential amplicon sizes and their overlap (e.g. refs 33, 34, 35). In ThermoAlign, a unique implementation of directed graph analysis was programmed for selecting the minimum number of primer pairs forming an amplicon tiling path with maximum coverage of the target region.

Finally, while many algorithms and tools have been developed for target-specific primer design, not all of these have reported on their performance in laboratory tests, such that the application of some tools remains unknown (Supplementary Table S1). This makes it difficult for end-users to assess the utility of a given tool amid a wide landscape of such tools. For those that have been tested in the laboratory, none have achieved 100% specificity in DNA amplification; however, reports have shown fairly good performance in a few species (yeast, human and soybean). Miura *et al*. (2005) evaluated their SDSS approach on the budding yeast genome and encountered some cases of off-target amplification. A study on a PrimerStation tool^27^ that also uses SDSS showed that all 10 primer pairs amplified specific products on human chromosome X. Reports on PRIMEGENS showed that ≈90% of the primer pairs produced a single amplicon^29^,^36^,^37^. Working in a genome scenario with a substantially greater repeat content (maize), we sought to develop a method that achieves near perfection in the automated design of primer pairs that amplify only on-target.

In this study, a genome-aware primer pair design algorithm embedded within a distributable tool called ThermoAlign was developed and validated. Priming specificity is determined based on genome-wide analysis of the hybridization for full-length primer-template interactions in the presence of non-complementary base pairings. For population studies, ThermoAlign is also capable of using prior information on the locations of genomic variants during the selection of primer pairs. In addition to the design of primer pairs for standard, single locus PCR amplification, a directed graph analysis method is used to design primer pairs forming amplicon tiling paths for targeted sequencing of large regions in a genome. Finally, the amplification specificity of primer pairs designed by ThermoAlign was validated on maize, one the most repetitive genomes (≈85% repetitive) for which a reference sequence is available^8^. ThermoAlign is expected to facilitate targeted genome sequence studies for any species.

## Results

**Figure 1:**
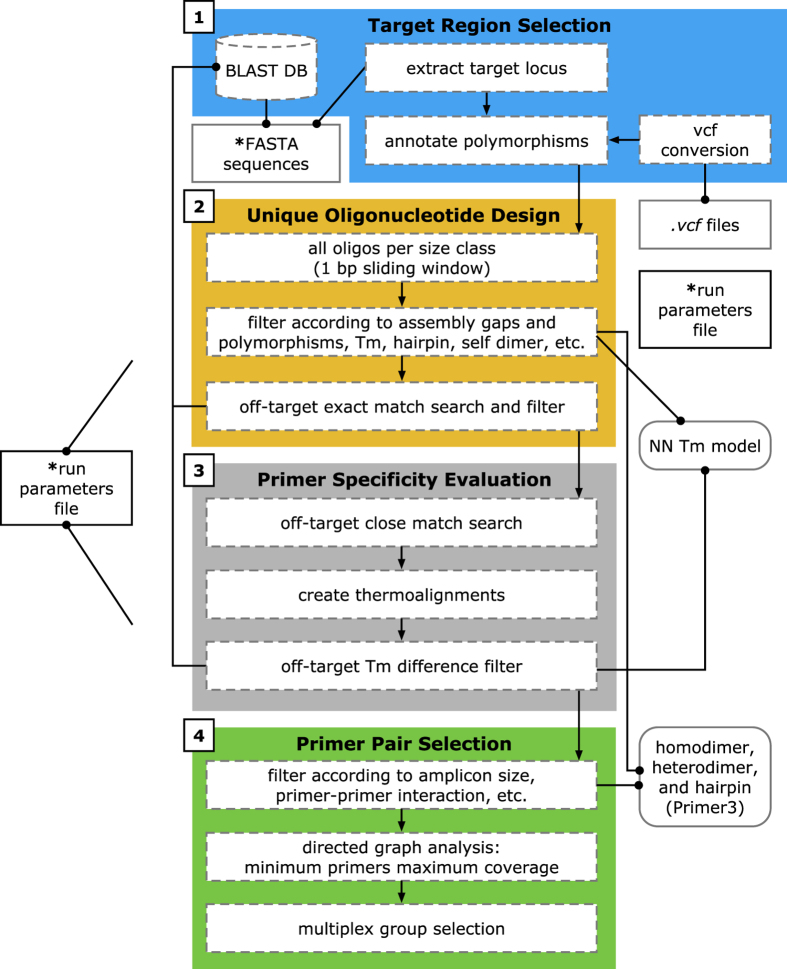
Schematic of the design strategy and workflow for ThermoAlign. A single run parameters file is used by all components of the pipeline. Colored boxes represent the four core modules of ThermoAlign, enumerated in their order of operation: (1) target region selection, (2) unique oligonucleotide design, (3) primer specificity evaluation, and (4) primer pair selection. Dashed boxes represent sub-routines within each of these modules and arrows depict their order of operation. The remaining elements are the database (reference genome sequence), external files (variant call format [*.vcf*] files and a run parameters file) and functions (nearest-neighbor model for the *T*_*m*_ of homodimer, heterodimer and hairpin interaction functions in Primer3). Connecting lines for these remaining components depict dependencies for the connected components (a filled dot is used to indicate the source from which information or a function is pulled). Required inputs for ThermoAlign are indicated by an asterisk.

**Figure 2:**
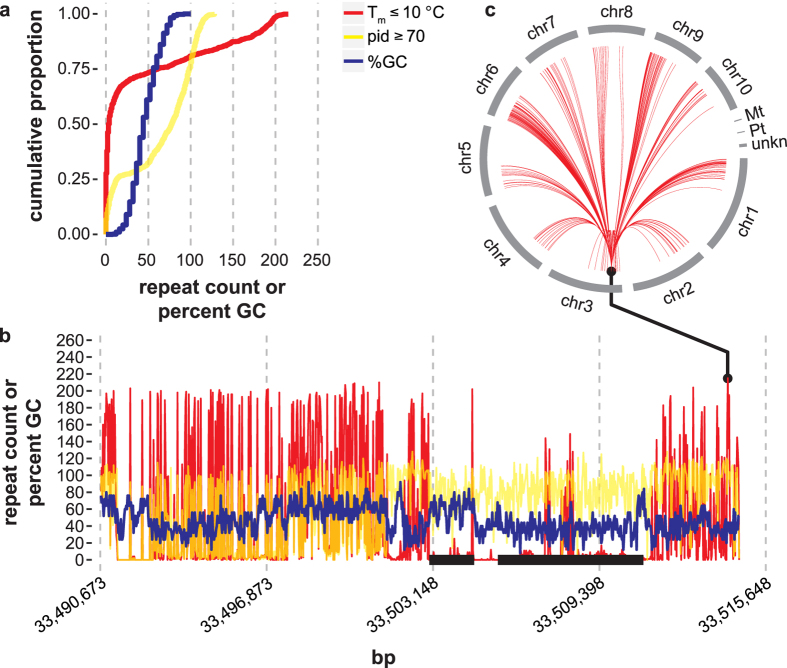
Repeat content and GC percentage across the 24 kb target region described in the text. The figure is based on analysis of each 25 bp sequence (26 bp sliding window) of the plus strand. For all subfigures, red lines show the number of thermoalignments with an off-target *T*_*m*_ within 10 °C of the corresponding on-target *T*_*m*_. Yellow lines (orange when overlapping with red) show the number of thermoalignments between a given primer and off-target sites with ≥70 percent identity (pid). Blue lines show the percent GC content. The search for off-target sites was based on BLASTn settings used in this study for priming specificity evaluation (see Methods), which had a maximum of 20 potential sites per pseudomolecule or a total of 260 possible sites. **(a)** Cumulative distribution of the number of repeats and percent GC content. **(b)** Genomic distribution of repeat content and GC percentage. The pseudomolecule coordinate of the 5′-nucleotide of each 25 bp sequence was used to position the plotted data. Black horizontal bars on the x-axis show the two genes in this region [left: GRMZM2G031364; right: GRMZM2G031239]. Among 25-mers in the region ≈73% would be predicted to have a misprime *T*_*m*_ within 10 °C of the primer *T*_*m*_. **(c)** The CIRCOS plot extends from a single primer in the region with the greatest number (n = 215) of predicted mispriming sites across the genome. Red lines of the CIRCOS plot connect the predicted mispriming sites on the pseudomolecules for chromosomes 1 to 10, the mitochondria (Mt), the plastid (Pt) and unmapped sequences (unkn).

A schematic of the ThermoAlign pipeline is shown in Fig. 1. In the following sections, results are presented pertaining to each module of the tool. A 24 kb target region of the maize genome (B73 RefGen_v3 Chr3:33490673..33514673) was used to demonstrate the pipeline and highlight features of ThermoAlign. Sixty-six percent of this region is annotated in the genome assembly as repeat-masked. Using the unmasked sequence and examining repetitiveness relevant to primer binding, 72% of the primers designed to this region would be predicted to produce off-target priming events at 1 to 215 sites for a given primer (Fig. 2). This same region, along with other segments of the genome, was used to test the amplification specificity of primers designed by ThermoAlign.

### Target Region Selection (TRS)

ThermoAlign produces an output file with summary information from the run (e.g. Supplementary File S1). Output for the 24 kb target region showed that it contained no gaps in the reference sequence assembly, 1,073 SNPs, 93 indels and 46% GC content.

### Unique Oligo Design (UOD)

The UOD algorithm was designed to identify every individual primer (not primer pairs) in a target region that is deemed favorable for PCR and has no identical matches elsewhere in the genome. For the 24 kb targeted region, among 184,145 total possible primers, 82,520 did not occur at sites containing polymorphisms in maize HapMap3^38^. Applying the full set of remaining UOD filters (for settings, see Supplementary File S2) resulted in the selection of 877 candidate primers.

The classification of the 82,520 primers into UOD filtering categories was examined to see which features had the largest effect on the removal of primers. This was split into two parts, starting with filters for primer sequence features and ending with filters for primer interactions (Supplementary Fig. S1). In terms of sequence features, 75,073 primers were filtered. Considering primers that were associated with only one sequence feature category, the A/T-end filter removed the largest number of primers (n = 9,217), comprising ≈50% of the collective set of primers that were specific to only one feature (Supplementary Fig. S1a). The A/T-end feature is a useful heuristic to eliminate primers with a greater potential for inefficient priming^39^. Optionally, the A/T-end filter or other filters may be excluded or re-parameterized to achieve a higher discovery rate of candidate primers, but this comes at the cost of increasing the computational time required for primer specificity evaluation (PSE; next section). For example, excluding the A/T-end filter from UOD resulted in 1,161 additional candidate primers (compared to 877 identified with the A/T-end filter applied), but this took approximately four times longer in runtime seconds for PSE.

The primer interaction filters, which were applied to the 7,447 primers that remained after filtering based on sequence features, included the occurrence of an exact match at an off-target site in the genome, homodimer *T*_*m*_, heterodimer *T*_*m*_ and hairpin *T*_*m*_^40^ (Supplementary Fig. S1b). This resulted in the filtering of an additional 6,570 primers, leaving 433 forward primers and 444 reverse primers with 136 being from the same position on the two strands.

### Priming Specificity Evaluation (PSE)

**Figure 3:**
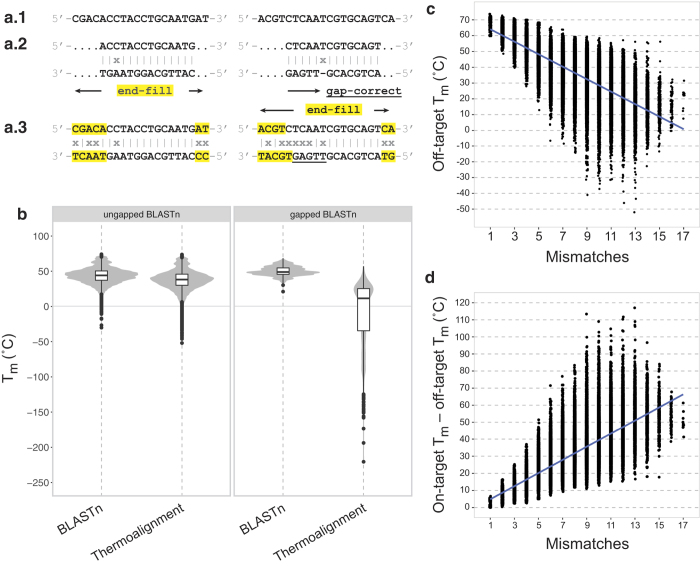
Creation and characteristics of thermoalignments compared to local alignments and the number of mismatches. **(a.1)** Examples of full-length primer sequences. **(a.2)** The top-ranking BLASTn high-scoring segment pair (HSP) alignment for two off-target sequences (bottom strand) is processed into a **(a.3)** thermoalignment by end-filling (ungapped BLASTn) or removing gaps and end-filling (gapped BLASTn) the original BLASTn HSP alignment. **(b)** For 877 candidate primers outputted by the UOD module for the 24 kb region described in the text, the *T*_*m*_ was calculated for each top-ranking BLASTn HSP alignment and the corresponding thermoalignment. **(c)** Using the subset of thermoalignments formed from ungapped BLASTn HSPs (n = 169,404 alignments), the plot shows the relationship between the off-target *T*_*m*_ for thermoalignments compared to the total number of mismatches. **(d)** Using the same subset of data in **(c)** the plot shows the difference between the on-target *T*_*m*_ and the off-target *T*_*m*_ of thermoalignments compared to the total number of mismatches.

A critical aspect of ThermoAlign is the algorithmic and quantitative approach used to characterize off-target hybridization sites. As part of the algorithm to determine the potential for mispriming, BLASTn alignments for each off-target match are edited into thermoalignments (full-length, ungapped primer-template alignments) that allow meaningful and accurate estimates of the *T*_*m*_ to be computed for a primer (Fig. 3). Native BLASTn alignments with ≥70% sequence identity (which are mostly truncated local alignments) had an average *T*_*m*_ that was 7 °C higher than their thermoalignment (Fig. 3b). However, the *T*_*m*_ for 10.8% (n = 18,834) of the BLASTn alignments was less than their thermoalignment (Fig. 3b). The range of the difference in *T*_*m*_ for BLASTn alignments compared to corresponding thermoalignments was −14 °C to 272 °C. Considering the relationship between the number of mismatches and *T*_*m*_, Fig. 3c,d showed that the number of mismatches, although correlated with thermoalignment *T*_*m*_, is not a suitable proxy for the potential of mispriming. Even in the presence of multiple mismatches, the *T*_*m*_ for binding at off-target sites can be at temperatures typical for PCR (e.g. >60 °C; Fig. 3c). Moreover, the off-target *T*_*m*_ may not always be sufficiently distant from the on-target *T*_*m*_ for specific priming to occur (Fig. 3d). For the data in Fig. 3d, ≈80% of the thermoalignments had an on-target *T*_*m*_ > 10 °C of the off-target *T*_*m*_.

### Primer Pair Selection (PPS)

From among the 877 oligonucleoltides that were expected to stably hybridize and specifically prime on-target within the reference genome, 2,818 combinations of primer pairs were found to be compatible for standard PCR. The parameter settings used for PPS (Supplementary File S2) included the requirement of a +10 °C difference in the *T*_*m*_ between the primer with the lower *T*_*m*_ of a given pair and the greatest off-target *T*_*m*_ for either of the two primers. Reducing this threshold can increase the discovery rate for primers, but one must consider a lower limit at which off-target amplicons would be likely to arise in actual PCR. When set to +6 °C, the number of primer pairs selected by the PPS module for the 24 kb region increased to 4,189. Adjusting this threshold along with the upper limit in the *T*_*m*_ range used for UOD can also increase the discovery rate. Increasing the *T*_*m*_ range by +5 °C (a change from 64–74 °C to 62–77 °C) while keeping a +10 °C maximum misprime difference led to the identification of 4,103 primer pairs via the UOD → PSE → PPS pipeline.

With the 877 primers from above, a directed graph method was used to identify the minimum number of primer pairs (shortest path) providing the maximum amount of coverage for the targeted region. The amplicon size range setting was a critical factor in the amount of coverage that could be achieved for the region examined here (Supplementary Table S2). Smaller amplicon size ranges led to relatively low coverage and the largest size ranges (≥15 kb) led to no coverage. Maximum coverage was achieved for amplicon sizes between 5 and 15 kb. However, recalling that the A/T-end filter resulted in the loss of over one thousand primers, excluding this filter increased the expected coverage from a maximum of 61.8% (with the filter) to 88.7% (without the filter).

### Empirical evaluation of priming specificity

**Figure 4:**
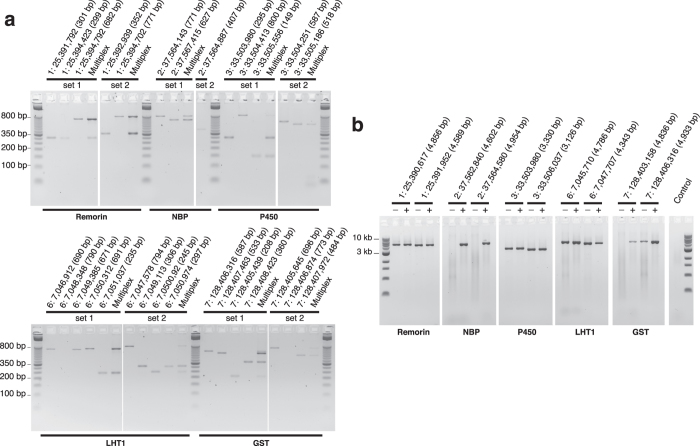
Empirical evaluation of ThermoAlign using standard and long-range PCR to tile five genes. The products from two additional genes amplified with standard PCR but not long-range PCR (as described in the text) are not shown. Labels indicate the chromosome number of the target locus, the forward primer start site and the expected size of the product. Details on each primer is available in Supplementary File S3. **(a)** Standard PCR products were quantified without post-PCR purification and approximately ≈7.5 ng were loaded into each well. For the two reactions that had no product, a volume equivalent to the average volume loaded was used. Multiplex reactions composed of primer pairs corresponding to each set for a given gene was loaded alongside the primers belonging to that same set. **(b)** Long-range PCR products from reactions without (−) and with (+) betaine. PCR products were quantified without post-PCR purification and ≈29 ng were loaded into each well. For the three reactions that had a no product, the same volume used for the corresponding betaine reaction was loaded into the well. For the negative control, the maximum volume used among all of the reactions was loaded into the well. The negative control was composed of master mix, primer pair TA_1_25390617_27_F and TA_1_25395472_24_R (Supplementary File S3) with no DNA template. Lanes with background smearing were associated with reactions that required a greater volume of the product be loaded to achieve a standardized amount of product across lanes.

Primer pairs designed by ThermoAlign were tested using standardized conditions for standard PCR and long-range PCR (see Methods section). For standard PCR, 46 primer pairs associated with seven genes located on six chromosomes of maize were tested (Supplementary File S3). Using the directed graph analysis method in PPS, these primer pairs were designed to tile from 1 kb upstream to 1 kb downstream of each gene. Thirty-eight of these primer pairs produced an amplicon, and for each of these a single specific amplicon of the expected size was observed; no off-target amplicons were detected for any of the primer pairs tested [Fig. 4a shows the results for 29 of the 46 primer pairs, two of which failed to amplify (6:7,048,348 and 7:128,406,874)].

ThermoAlign integrates MultiPLX^41^ while customizing the input and output to obtain two groups of multiplexes compatible with the amplification of overlapping tiling paths. For each of the seven targeted genes tested using standard PCR, under the “normal” stringency settings, MultiPLX identified multiplexes with no more than two primer pairs (the possibility existed to combine as many as five primer pairs). The amplicons produced using multiplex PCR were generally consistent with those produced by each primer pair individually (one primer pair in one multiplex set failed in the multiplex reaction) and no alternative amplicons were observed (Fig. 4a).

For five of the seven genes mentioned above, 0.1–5.0 kb amplicon tiling paths were designed for each gene (independent of the standard PCR primers; Supplementary File S3) and tested using long-range PCR. For each gene, two primer pairs were identified that would tile across the full length of the gene (one exception: with the settings used, primer pairs were not found that would cover the entire P450 gene on chromosome 3). Similar to standard PCR, not all ten primer pairs produced an amplicon, but the seven that did produced a single prominent amplicon of the expected size (Fig. 4b). For long-range PCR amplicons that failed to amplify or had low yield, more of the reaction product was loaded into the gel in order to normalize the products for comparison. This showed some background smearing that was greater than the negative control, suggesting some amount of random off-target amplification had occurred during long-range PCR (potentially due to mega-primer amplification^14^).

Because of the dependency of a reference genome for primer design and that some standard PCR and long-range PCR reactions failed to produce amplicons, we questioned whether these failed reactions were due to inaccuracies in the sequence assembly. Under the assumption that long-range PCR primer pairs that produced a specific amplicon of the expected size were an indication of an accurate assembly, the production of standard PCR amplicons nested within these long-range PCR amplicons was used to address this question.

Twenty-nine standard PCR primer pairs were designed to the same five genes tested by long-range PCR and were nested within at least one of the expected long-range PCR amplicons. Some of the standard PCR amplicons were nested within overlapping sections of two long-range PCR amplicons where one of the primer pairs produced a product and the other did not. Excluding those standard PCR primer pairs from consideration, one out of 21 of the standard PCR primer pairs failed to produce an amplicon in regions where an amplicon was produced by long-range PCR. In contrast, all five standard PCR primer pairs produced an amplicon in regions where no amplicon was produced by long-range PCR. The association between successful and failed reactions for standard and long-range PCR was not significant (Fisher’s Exact Test, *p* = 1.0), which failed to implicate assembly errors as the cause for PCR failures.

Considering the possibility that the sequence composition of the primers or amplification target affected success^14^, the addition of betaine to the reactions resulted in all 10 long-range PCR primer pairs producing a specific product of the expected size (Fig. 4b). Subsequent testing of standard PCR primer pairs with betaine resulted in the recovery of a single specific amplicon for the two nested pairs that had failed in the absence of betaine, in addition to four primer pairs from the original set of 46. However, these products amplified poorly (data not shown). Additional PCR optimization could potentially improve the amplification efficiency of these primer pairs. The amplicons of reactions that were recovered by the addition of betaine for long-range PCR had a higher median GC content by 3.2 percentage points for the primers and 7.8 percentage points for the expected amplicons (B73 reference genome sequence). Similarly, standard PCR reactions that were recovered using betaine (considering all 46 of the primer pairs) had a higher median GC content for the primers (3.7 percentage points) and expected amplicons (19.7 percentage points).

To confirm that the amplicons corresponded to the targeted loci, nine of the ten long-range PCR products in Fig. 4b were pooled and sequenced by single molecule, real-time sequencing. A primer-based clustering and sequence analysis approach generated exactly nine consensus sequences with perfect identity to the expected sequence (Table 1; Supplementary File S4).

## Discussion

Through the current era of high-throughput sequencing, the design of oligonucleotides has remained a fundamental need for genome science research and applications. Models for designing hybridization and priming oligonucleotides can shed light on the understanding of oligonucleotide-template interactions while providing the design products for probing genomes. Two key aspects are whether hybridization and priming will occur and whether these events are specific to the targeted sequence. In this study, specificity of primer pair amplification was addressed in the development of ThermoAlign for the automated design of priming oligonucleotides. ThermoAlign assesses specificity by computing the nearest-neighbor thermodynamics of hybridization^20^ for full-length oligonucleotide-template interactions across the genome. Computationally empirical results suggested there is no sufficient substitute for this. Approaches that rely on sub-sequences (characterized here based on local BLASTn alignments; Fig. 3b) of a primer-template interaction or the number of mismatches in a thermoalignment (Fig. 3d) for specificity evaluation are expected to have a greater risk of selecting primers that would hybridize at off-target sites and a lower discovery rate for identifying suitable primers.

Examination of genome-wide thermoalignments for primers in a 24 kb region of the maize genome showed that the proportion of repeat content in the context of a primer space (25 bp) was greater than that based on transposable element repeat masking. This emphasizes the importance of a genome-aware primer specificity evaluation process. Applying this approach to the repetitive genome of maize resulted in a single and specific amplicon of expected size for every primer pair that produced a product by standard PCR, without any modifications to the reaction chemistry (Fig. 4). Long-range PCR gave essentially the same results. Although some of those reactions produced some random background amplification, a single prominent amplicon was observed, and the expected sequence was obtained by sequencing. We have continued to observe near-perfect specificity for an additional 50 functional primer pairs tested on multiple samples of maize (data not shown). Our results demonstrate the accuracy of the nearest-neighbor thermodynamics model and the principles embedded in ThermoAlign for primer design. The results of this study lend support to prior studies that have developed chemistry-conditioned nearest-neighbor thermodynamic models to estimate energy parameters for DNA duplexes that may include non-complementary bases^21^,^22^,^23^,^24^,^25^,^26^,^42^.

**Figure 5:**
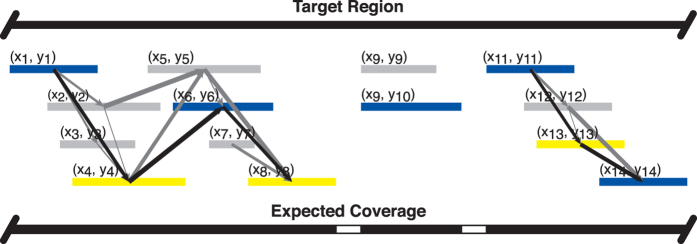
Directed graph analysis approach used in ThermoAlign to identify primer pairs for an amplicon tiling path at a targeted region. *x*_*i*_ and *y*_*i*_ represent forward and reverse primers respectively. Nodes correspond to expected amplicons (depicted as colored bars), which are ordered by their base pair coordinates. Directed edges (lines with an arrowhead) are drawn between overlapping amplicons, which in this case forms two subgraphs. Edge weights (depicted by the thickness of the lines representing edges; here, thicker lines represent the smaller weighted edges that would be selected) are computed based on the cumulative coverage and amount of overlap. Dijktra’s shortest path algorithm is applied to each subgraph to identify the primer pairs comprising the minimum tiling path of amplicons (black colored edges). As portrayed by the blue and yellow coloring, two groups of non-overlapping primer pairs are designated for multiplex consideration. The expected coverage for this example minimum tiling path is indicated by black (covered) and white (gaps) fill.

ThermoAlign is an elaborate pipeline that includes a suite of modules with a number of unique elements (Supplementary Table S1). This includes the creation of thermoalignments to obtain accurate estimates of hybridization energies and a graph analysis approach for automated identification of amplicon tiling paths for resequencing studies. Therefore, ThermoAlign can be used not only to design primer pairs for standard, single-locus PCR applications, but also for resequencing studies of targeted regions that cannot be captured with a single primer pair. For population studies, a polymorphism-aware feature allows primers to be designed to monomorphic regions of the genome. However, this will be limited by the extent of prior information pertaining to the population(s) under study. To increase the efficiency of laboratory efforts to generate amplicons for resequencing, primer pairs constituting amplicon tiling paths are passed to the software MultiPLX^41^ and organized into multiplex compatible sets (Fig. 5). Finally, as a matter of extensibility, the graph analysis method used in ThermoAlign can be re-structured in terms of how edge weights are computed. For instance, as it is sometimes desirable to obtain more similarly sized amplicons for sequencing, we foresee that edge weights could be adjusted according to a probability function centered on a desired amplicon size.

This study demonstrates that nearest-neighbor estimation of *T*_*m*_ on thermoalignments is a robust solution for target-specific primer design. ThermoAlign requires minimal computational power and quickly identifies suitable primer pairs for routine applications targeting a single locus of a few hundred to a few thousand base pairs. This makes the tool useful for routine PCR applications. However, *de novo* searches using BLASTn may be suboptimal and PSE requires greater computational resources for large target regions with run parameterizations having broad search settings. This is despite having optimized the settings for BLASTn such that the search was limited to obtaining sufficient information for PSE (see Supplementary information and Supplementary Fig. S2). Clocking the speed of each component showed that, not surprisingly, the genome-wide search for non-exact matches in the PSE module is a key component to focus on for reducing run times (Supplementary File S1). Therefore, other alignment search methods or a database of pre-computed *T*_*m*_ (e.g. ref. 27) could be considered for reducing the run time. Furthermore, the BLASTn approach may not identify all mispriming sites for a given primer because it does not allow for mismatches with the sequence used to seed an alignment. Ideally, the *T*_*m*_ of every possible thermoalignment across the genome would be computed for every candidate primer considered.

Another critical aspect that remains to be addressed by primer design tools is determining the likelihood for successful amplification. This has received recent attention, leading to insights into how this might be predicted *in silico* from oligonucleotide and template features^14^,^43^,^44^. Methods for predicting amplification success could be readily integrated into ThermoAlign. By comparing the success of standard PCR primer pairs nested within long-range PCR primer pairs, we observed that assembly errors were not likely to have caused failures to amplify the regions tested in this study. The conversion of failed reactions into successful ones following the addition of betaine to PCR reactions suggested secondary structures as the likely cause of failure. Betaine has been shown to improve the amplification of GC rich regions by reducing the formation of secondary structures, making the template more accessible to DNA polymerase^45^,^46^. Indeed, as it has been widely documented, this study found an association between high GC content of primers and the target sequences with failure to amplify by PCR in the absence of betaine.

ThermoAlign is founded upon thermodynamically relevant sequence alignments or thermoalignments for estimating hybridization energies of oligonucleotides across a sequenced genome. This results in the design of target-specific primer pairs for PCR and tiled amplicon resequencing. We have demonstrated that the selection of such primer pairs is fully automatable and was applicable to a species (*Zea mays* subsp. *mays*) with ≈85% repeat content in its genome. The model used to extend local alignments was developed with priming in mind, but an alternative model could be developed for hybridization probes, which would require evaluation of the *T*_*m*_ for bidirectional gap-adjusted end-filling models (if the BLASTn algorithm is maintained). The modular design of ThermoAlign and open source license provides an extensible resource for continued development that can be adapted for a range of other applications including the design of hybridization oligonucleotides and gene editing techniques. The applications for ThermoAlign currently range from standard PCR assays to long-molecule resequencing. Multiplexed libraries containing pools of separate, long-range PCR products, such as those tiling across a target region and amplified on multiple individuals, can be sequenced on current parallel sequencing platforms, placing ThermoAlign as a tool for studies using high-throughput sequencing.

## Methods

### ThermoAlign pipeline

A thermodynamics-based, genome-wide specificity evaluation approach for designing priming oligonucleotides was developed. The approach consists of four stages: (1) selection of a targeted sequence in a genome and masking of variant sites; (2) extraction of all possible oligonucleotides from the target region that are unique and identification of those expected to stably hybridize with the DNA template; (3) evaluation of the mispriming potential for candidate primers; and (4) identification of all suitable primer pairs including a minimum set that provides maximum coverage of the targeted region (Fig. 1). Users can modify run parameters corresponding to each of the ThermoAlign modules in a single settings file (parameters.py; see the GitHub repository referenced in the Availability section below). The ThermoAlign pipeline was developed in Python 2.7.5 and evaluated on a machine equipped with dual 64-bit 2.1 CPU GHz AMD Opteron processor 6172, using 5 of its cores, running on Fedora release 19 and 23 OS.

### Target region selection (TRS)

The TRS module uses external files and custom pre-processing scripts to create a BLAST database and modify the targeted sequence for subsequent filters in the pipeline. For a given FASTA formatted reference genome sequence, the sequence for a target region is extracted based on user provided coordinates.

#### External files

ThermoAlign minimally requires FASTA formatted files for a reference genome. This is used to extract the targeted sequence for designing oligonucleotides and to create a BLAST database to search for off-target priming sites. Standard variant call format (*vcf* v4.0 and v4.1; https://vcftools.github.io/index.html) files based on the same coordinate system of the reference genome sequence may be optionally used for polymorphism-aware primer design.

#### BLAST database

The sequences for each pseudomolecule are required as separate FASTA files using a specific naming convention. Formatting details are provided at the GitHub repository cited in the Availability section below.

#### Pre-processing *vcf* files (optional)

A custom script (vcf_conversion.py) is used to convert input *vcf* files into files compatible with ThermoAlign scripts containing only the genome coordinates and corresponding variant information. These pre-processed, smaller sized data files enable more efficient parsing and annotation of polymorphic bases in the target region.

#### Polymorphism annotation (optional)

Nucleotides at variant sites are converted to “n” [single nucleotide polymorphism (SNP)] or “N” (indels), which are later used as indicator characters to filter primers associated with these sites. For this study, maize HapMap3^38^ was used for prior variant information. Although indels may comprise multiple nucleotides, maize HapMap3 encodes indels as a single nucleotide position without information on the start and stop positions or length of the indel. Therefore, the individual nucleotide at the corresponding position for each indel in HapMap3 was converted to an N.

#### Assembly gap consideration

The maize reference genome contains strings of 100 and 1000 “N” characters to represent gaps for sequences from individual clones and between assembly scaffolds, respectively, which could be distinguished from SNP and indel locations encoded as a single “n” or “N.” In terms of coordinates, each and every “n” and “N” character were treated as having a position (as was the case for the original reference genome coordinates).

### Unique oligonucleotide design (UOD)

The UOD algorithm was designed to extract candidate oligonucleotides from the target region and characterize their properties relevant to hybridization and priming. Across a user-defined range of oligonucleotide lengths, and using a 1 bp sliding window for each length, every possible oligonucleotide sequence was extracted from both the positive and negative strands. A series of filters were applied to the entire set of oligonucleotides in the following order of operations.

#### Flanking primers (user-specified)

UOD can optionally design primers that flank a given target locus. Forward primers with their 3′-end position that is less than or equal to the locus start and reverse primers with their 3′-end position that is greater than or equal to the locus stop are designed. Flanking primers are designed according to a specified flanking size.

#### Base composition (polymorphism filter optional)

Oligonucleotides containing any character other than A, C, G or T were filtered (this removed primers containing any “n” or “N” character). This ensured that oligonucleotides corresponding to polymorphic sites and gaps in the sequence were avoided. However, the filtering of oligonucleotides with polymorphic sites is optional.

#### GC content (user-specified)

Oligonucleotides that fell outside a defined range of percent GC were filtered. In this study, a range of 40–60% GC was used.

#### A/T-end (optional)

For primers, a single G or C nucleotide at the 3′-end helps to stabilize binding near the site of extension, which can reduce the possibility of “breathing” and improves priming efficiency^39^. Therefore, primers ending in an A or a T base could optionally be filtered. In this study, the filter was applied.

#### GC clamp (optional)

Oligonucleotides with more than three G or C nucleotides within the last five bases were filtered. This should help minimize mispriming at GC-rich binding sites^39^.

#### Repeating nucleotides (user-specified)

Oligonucleotides with four or more mononucleotide or dinucleotide repeats were filtered.

#### Melting temperature (user-specified)

An important component of the pipeline is estimation of melting temperature (*T*_*m*_). The nearest-neighbor estimator was used as described by Santalucia and Hicks^21^:


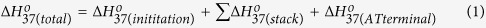



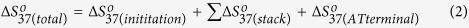



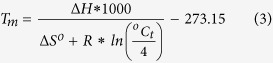


where Δ*H*^0^ and Δ*S*^0^ are enthalpy (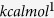) and entropy (*e.u.*) respectively. We refer the reader to Santalucia and Hicks, 2004 for further details on equations, units and definitions. To obtain *T*_*m*_ for different reaction conditions, the *Na*^+^ equivalent of monovalent and divalent ions were calculated according to Von Ahsen, 2001^42^, which were used to adjust Δ*S*^0^(*total*) [equation 5 in Santalucia and Hicks, 2004^21^], where:


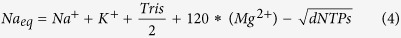


however, if 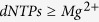, then


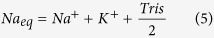



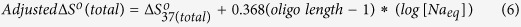


Primers that fell outside the specified range in *T*_*m*_ were filtered. In this study, a *T*_*m*_ range of 64–74 °C was used for primer design.

#### Self-primer interactions (user-specified)

The Primer3 thal.c library functions^40^ were used to compute hairpin and self-dimer *T*_*m*_ values (functions: primer3.calcHairpin and primer3.calcHomodimer; Primer3 release 2.3.7). A primer was filtered if either of these *T*_*m*_ values were within 20 °C of the corresponding primer-DNA hybridization *T*_*m*_.

#### Exact match (user-specified)

The remaining candidate primers were screened by BLASTn (ThermoAlign default optimized settings: e_value: 30000; word_size: smallest primer length; gapopen: 2; gapextend: 2; reward: 1; penalty: -3; max_target_seqs 2; max_hsps 2) to determine if at least one off-target exact match occurs within the reference genome, in which case the primer was filtered. Note: setting the max_target_seqs to 2 and max_hsps to 2 provides the quickest search that ensures at least one match other than the target priming site can be found. Setting either of these less than 2 could cause ThermoAlign to fail in identifying off-target exact matches.

### Priming specificity evaluation (PSE)

Following the identification of unique primer sequences with adequate properties for amplification, a more computationally intensive algorithm, PSE, was used to evaluate specificity of the remaining primers as follows:

#### Nearly identical match

BLASTn (ThermoAlign default optimized settings: e_value: 30000; word_size: 7; gapopen: 2; gapextend: 2; reward: 1; penalty: -1; perc_identity: 70; max_target_seqs: 13; max_hsps: 20) was used to identify off-target sites across the reference genome that partially match candidate primers, considering that these could serve as mispriming sites.

The parameters used for PSE required some consideration. A low stringency e-value cutoff was used to account for the higher probability that short sequences would have matches in the database. The word size value is an important parameter. A perfect match across the word size is required to seed a BLASTn alignment, but the PSE module seeks to find imperfectly matching sequences. The smallest word size available for BLASTn was used to provide the maximum sensitivity for discovery of potential off-target sites. Gapopen and gapextend scores of 2, a reward score of 1 and a penalty score of 1 were used to support the alignment of divergent sequences^47^. Selecting alignments with a percent identity of 70% or more ensures a thorough evaluation of potential off-target priming sites. Figure 2 shows that primers with ≥9 mismatches with off-target sites have a *T*_*m*_ that is at least 10 degrees less than the on-target site. The maximum number of target sequences (max_target_seqs) corresponded to the total number of FASTA sequences in the reference genome database (for maize this included 13 sequences comprising nuclear chromosomes 1–10, the mitochondrial genome, the chloroplast genome and a tethered set of unmapped sequences). To avoid extensive searches, based on a preliminary PSE algorithm it was found that 20 high-scoring segment pairs (HSPs) per target sequence identified most sites within the maize genome that candidate primers might hybridize with (i.e. this was a sufficient search space to provide information for filtering a primer; data not shown). While this max_hsps setting was used for designing primers in this study, an updated analysis suggested a max_hsps setting of 50 may be more appropriate for future work (Supplementary Fig. S1). The low complexity filter was disabled to allow for an exhaustive search.

#### Thermoalignments

Since the alignment algorithm of BLASTn maximizes the scores on local alignments, it can return truncated alignments between the primer and template sequences. For each truncated alignment, given the alignment start and stop coordinate and the length of the primer, an end-filling algorithm was developed that extends the alignment to the full length of the primer sequence (Fig. 3). This was done in a manner that maintains strandedness. Given the strand-specific 5′ and 3′ coordinates to which the full-length primer is expected to align, the corresponding region of the genomic sequence was extracted. In some cases, BLASTn may introduce gaps in the alignment while real primer-template interactions are gapless. To address this, strand-specific algorithms were developed to create full-length, ungapped primer-template alignments relevant to priming oligonucleotides (Fig. 3). Gaps introduced in the primer sequence (deletions in the primer relative to the template) were corrected by shifting the bases in the primer that were 5′ of the gap toward the 3′-end of the primer, and then discarding the resulting overhanging nucleotides at the 5′ end of the template sequence. Gaps introduced in the template sequence (insertions in the primer relative to the template) were corrected by shifting the bases in the template that were 3′ of the gap toward the 5′ end of the template, and then extending the template sequence corresponding to the resulting overhang at the 5′ end of the primer. This algorithm was designed to maximize the number of complementary bases toward the 3′ end of the primer, i.e., the site of primer extension; a different approach could be considered if ThermoAlign is extended for the identification of hybridization probes. The *T*_*m*_ was calculated for each ungapped full-length primer-template alignment which we refer to as a thermoalignment.

#### Misprime *T*_*m*_

Using the same formulae outlined under the melting temperature calculations, the *T*_*m*_ was calculated for each thermoalignment between a primer and its off-target template locations. All of these thermoalignments contain at least one mismatch (oligonucleotides with exact matches at off-target sites were filtered in UOD). Therefore, parameters for G/T mismatches^25^, G/A mismatches^24^, A/C mismatches^23^, C/T mismatches^22^ and A/A, C/C, G/G, T/T mismatches^26^ were used for the computation of *T*_*m*_.

#### Maximum misprime *T*_*m*_ (user-defined)

For each candidate primer, among each potential off-target hybridization site in the genome, the maximum *T*_*m*_ and the 90^*th*^ percentile *T*_*m*_ of thermoalignments are identified. Primers are selected if their on-target *T*_*m*_ is greater than their maximum off-target *T*_*m*_ as defined by a user-defined threshold. In this study, a +10 °C threshold was used.

#### 3′-end mismatch

Primers with a 3′-terminal mismatch at a biding site are not expected to produce off-target synthesis products under standard PCR conditions^30^. Therefore, off-target thermoalignments that included a mismatch at the 3′-terminal nucleotide were not considered in PSE. In addition, the proportion of thermoalignments with any number of mismatches within the last five bases at the 3′-end were recorded in output but not used for filtering.

### Primer pair selection (PPS)

Once primers were determined by the TRS → UOD → PSE pipeline, primer pairs were identified using the following criteria.

#### Amplicon length (user-specified)

Amplicon length was calculated as the distance between the 5′-ends of the forward and reverse primers. Primer pairs that would produce an expected on-target amplicon within a user-defined range in length were maintained. In this study, different settings were used for different experiments.

#### Assembly gaps and indels (including an optional gap filter)

The presence of assembly gaps (represented as 100 or 1,000 nucleotide runs of an N) and indels (HapMap info) in the expected amplicon for a primer pair is indicated in the output. For this study, primer pairs expected to produce amplicons containing gaps in the reference genome were filtered while amplicons containing indels were not.

#### *T*_*m*_ difference (user-specified)

Primer pairs differing by 10 °C or less in *T*_*m*_ were selected. Although it seems inappropriate to choose such a large difference in *T*_*m*_ between primer pairs, so long as the *T*_*m*_ of each primer is sufficiently greater than the maximum misprime *T*_*m*_ of either primer then the primer pair should still be capable of locus specific amplification, using an annealing temperature appropriate for the primer with the lowest *T*_*m*_.

#### Maximum misprime *T*_*m*_ (user-specified)

The primer with the lowest *T*_*m*_ in a given primer pair must be greater than the maximum misprime *T*_*m*_ of both primers in the pair according to a user defined threshold value. In this study, a 10 °*C* threshold was used.

#### Heterodimer ΔG (user-specified)

The Primer3-py API 0.5.0 (https://github.com/libnano/primer3-py) was used to compute heterodimer ΔG values. Primer pairs with less than −2000 kcal/mol ΔG values were filtered in this study. We note that during our development phase, using what had been the most current version of Primer3 (v. 2.3.6), we discovered that the entropy and enthalpy estimates for dimers depended upon the input order of the two primer sequences. In v. 2.3.7 the results are no longer a function of the primer input order.

#### Amplicon features

The amplicon sequence corresponding to each primer pair was summarized in terms of features such as GC content, presence of indels and assembly gaps.

#### Minimum primer pair set identification

In situations where amplification of a long section of the genome is the objective, a set of primer pairs producing a tiling path of overlapping amplicons may be designed. We employed a graph analysis strategy to identify the minimum set of primers to achieve maximum coverage of the target region, along with grouping of the resulting primers into multiplex compatible sets. For a given target region, a directed graph was constructed for all possible primer pairs (Fig. 5). Each primer pair (potential amplicon) was represented as a node. Directed edges between any two nodes (*N*_*i*_, *N*_*j*_) were made for overlapping amplicons, given that the node *N*_*i*_ starts (5′ position of forward primer) at a position before that of the overlapping node *N*_*j*_; and *N*_*j*_ ends (5′ position of reverse primer) after the corresponding end position of *N*_*i*_. To handle instances in which a target region contained gaps because no pass-filter primer pairs could be identified to produce a continuous set of overlapping amplicons, subgraphs were created for the target region. Edge weights (*E*_*i*_) were calculated based on the cumulative sequence coverage for each pair of overlapping amplicons (*C*_*jk*_), after penalizing for the amount of overlap between them (*O*_*jk*_): *E*_*i*_ = *C*_*jk*_ − *O*_*jk*_. The highest possible edge weight for a given graph (*M*_*g*_) was subtracted from the original edge weights to adjust the scale, such that the original higher scored edges were transformed into the lower scored edges for a given subgraph: *W*_*i*_ = *M*_*g*_ − *E*_*i*_. From the directed graph with adjusted edge weights (*W*_*i*_), the shortest path was identified based on Dijkstra’s shortest path algorithm^48^,^49^. The creation of the graph and identification of the shortest paths was implemented using the NetworkX 1.11^50^ python package.

The directed graph based approach identifies the minimum number of primers that gives the maximum coverage of a targeted genomic region in a computationally efficient manner. There was one exception in which we abandoned graph analysis. When two or more amplicons of different sizes covered the same genomic segment and started or ended with the same primer and did not overlap with other segments (i.e. amplicons were not offset on both sides), the single amplicon that gave the maximum coverage was used. Together, these strategies ensured that the minimum set of primer pairs with the maximum coverage for a given target region are identified.

The resulting primer pairs were split into two sets where each set contained primer pairs that would not produce overlapping amplicons (Fig. 5). The separate sets were evaluated as to whether they could form multiplex sets using MultiPLX^41^. Attempting to multiplex primer pairs that produce overlapping amplicons in one set would lead to PCR production of undesirable or small amplicons from primers at the ends of overlapping segments; due to their smaller size their production could dominate the reaction.

#### ThermoAlign output

ThermoAlign produces the following primary output files: (i) a text file summarizing pipeline settings and results; (ii) a text file for ordering primers; (iii) a text file with information about the primer pairs and resulting amplicon features; (iv) a text file with the minimum primer pairs giving maximum coverage that are also grouped into compatible multiplex sets; and (v).*bed* formatted files of the primers for further analysis and visualization. For further details on these and additional output files see the README documentation at the GitHub repository).

### PCR validation

Standard PCR was performed using *Taq* DNA polymerase with standard *Taq* (Mg-free) Buffer (New England Biolabs, Ipswich, MA; Cat#M0320). Reactions with a final concentration of 1X standard *Taq* (Mg-free) reaction buffer, 1.5 mM MgCl_2_, 200 μM dNTPs, 0.1 μM forward primer, 0.1 μM reverse primer and 0.625 U *Taq* were combined with 20 ng of gDNA and brought to a volume of 25 μL with molecular-grade water. Some reactions were supplemented to include a final concentration of 1 M betaine. Amplification was carried out on an Eppendorf Mastercycler pro S with the following conditions: 5 min at 95 °C; 30 cycles of 20 s at 94 °C, 20 s at 4 °C below the minimum *T*_*m*_ for the primer pair as computed by ThermoAlign and 1 min at 68 °C; 5 min at 68 °C; and a hold at 4 °C. PCR products were assayed by electrophoresis in a 3% TBE gel (Bio-Rad, Hercules, CA; Cat#161-3040) and imaged using a FluorChem HD2 with AlphaView SA v3.4.0.

Long-range PCR was performed using GoTaq Long PCR Master Mix (Promega, Madison, WI; Cat#M4021). Reactions with a final concentration of 1X GoTaq Long PCR Master Mix, 0.1 μM forward primer and 0.1 μM reverse primer were combined with 25 ng gDNA and brought to a volume of 16 μL with molecular-grade water. Some reactions were supplemented to include a final concentration of 1 M betaine. Amplification was carried out in an Eppendorf Mastercycler pro S with the following conditions: 2 min at 94 °C; 30 cycles of 30 s at 94 °C, 5.5 min at 65 °C (following a 1 min/kb guideline based of the longest expected amplicon in the set); 10 min at 72 °C; and a hold at 4 °C. PCR products were assayed via electrophoresis in a 1% TBE gel (Bio-Rad; Cat#161-3038) and imaged using a FluorChem HD2 with AlphaView SA v3.4.0.

### SMRT sequencing and analysis of long-range PCR amplicons

Amplicons for each long-range PCR primer pair were cleaned with SPRIselect (Beckman Coulter, Brea, CA; Cat#: B23319) at a ratio of 1:1 (beads:sample). Fragment analysis confirming the expected size amplicons was performed using a bioanalyzer (data not shown). Cleaned samples were then quantified using PicoGreen dsDNA quantitation assay (ThermoFisher, Waltham, MA; Cat#P7589) and pooled so that each amplicon was at 0.4 nM. Additionally, the same set of samples was pooled following^51^ to account for differences in molar mass. For each pool, approximately 900 ng of pooled amplicons was submitted to University of Delaware’s Sequencing and Genotyping Center (http://www1.udel.edu/dnasequence/Site/Home.html) for SMRTbell library construction and sequencing on one SMRTcell per library using the PacBio RSII.

PacBio SMRT raw reads were clustered by the primer sequences. The number of circular consensus sequences per cluster were recorded. Each cluster of reads was error corrected using Quiver in PacBio SMRT analysis V2.3. LAA protocol to get their consensus sequences. These sequences were aligned to the B73 reference genome using BLASTn (Expect threshold: 0.0001, word size: 15, match/mismatch scores: 1,−2).

### Availability

ThermoAlign is released under a GNU GPLv3 open source license at https://github.com/drmaize/ThermoAlign and has been packaged as a distributable tool in Docker containers. The following Docker images are available: (i) TA_1.0.0_d is a general distributable version which requires user supplied files; (ii) TA_1.0.0_s is a sample run version containing a small set of sample files that can be used to test ThermoAlign; (iii) TA_1.0.0_Zm3 is a maize ready version. Instructions for using ThermoAlign and running these containers can be found at the GitHub repository.

## Additional Information

**How to cite this article:** Francis, F. *et al*. ThermoAlign: a genome-aware primer design tool for tiled amplicon resequencing. *Sci. Rep.* **7**, 44437; doi: 10.1038/srep44437 (2017).

**Publisher's note:** Springer Nature remains neutral with regard to jurisdictional claims in published maps and institutional affiliations.

## Supplementary Material

Supplementary Information

## Figures and Tables

**Table 1:** Results from BLASTn alignment of error corrected PacBio consensus sequences to the B73 genome.

Amplicon label	Chr.	Start position	Stop position	Alignment length (bp)	Percent identity	CCS depth^1^
1: 25,390,617 (4,856 bp)	1	25,390,617	25,395,472	4,856	100%	56
1: 25,391,952 (4,589 bp)	1	25,391,952	25,396,540	4,589	100%	2172
2: 37,562,840 (4,602 bp)	2	37,562,840	37,567,441	4,602	100%	3988
2: 37,564,580 (4,954 bp)	2	37,564,580	37,569,533	4,954	100%	1384
3: 33,503,980 (3,330 bp)	3	33,503,980	33,507,309	3,330	100%	2718
3: 33,506,037 (3,126 bp)	3	33,506,037	33,509,162	3,126	100%	732
6: 7,045,710 (4,786 bp)	6	7,045,710	7,050,495	4,786	100%	2031
6: 7,047,707 (4,343 bp)	6	7,047,707	7,052,049	4,343	100%	254
7: 128,406,316 (4,933 bp)	7	128,406,316	128,411,248	4,933	100%	4727

^1^Circular consensus sequence (CCS) is PacBio terminology for consensus sequences formed from subreads per zero-mode waveguide. The sequence used for BLASTn was the consensus of all CCSs for a given amplicon).
