# Supplementary material for: ThermoAlign: a genome-aware primer design tool for tiled amplicon resequencing
Source: Sci Rep. 2017 Mar 16;7:44437. doi: 10.1038/srep44437 (PMC5353602; doi:10.1038/srep44437)
Supplement: Supplementary Information [file srep44437-s1.pdf]

ThermoAlign: a genome-aware primer design tool for tiled amplicon resequencing

Felix Francis, Michael D. Dumas and Randall J. Wisser

Supplementary information

Supplementary Tables

Table S1. Comparison of ThermoAlign to related primer design tools.

			Specificity evaluation								
Name	Primer design[1]	Probe design	Genome aware	NN model[2]	Full-length alignment[3]	Off-site primer pair	Search algorithm	Amplicon tiling path	Multiplex grouping	Lab validation	Reference[4]
Primer3	Y [C]	Y [c]	N	N	Y	N	BLAST;<br>Smith Waterman	N	N	N	Untergasser *et al.*, 2012[1], [a]
GENOMEMASKER	Y [S]	N	Y	N	N	Y	k-mer counts;<br>e-PCR	N	N	N	Andreson *et al.*, 2006[2], [a]
MFEprimer-2.0	N	N	Y	Y	N	Y	Indexed<br>database search	N	N	N	Qu *et al.*, 2012[3], [a]
PRIMEGENSw3	Y [C,S]	Y [C,S]	Y	Y	N	Y	MegaBLAST	N	N	Y	Srivastava *et al.*, 2011[4], [a];<br>Kushwaha *et al.*, 2015[5], [b]
Primer-BLAST	Y [S]	Y [S]	Y	N	Y	Y	BLASTn;<br>Needleman-Wunsch	N	N	N	Ye *et al.*, 2012[6], [a]
SDSS	Y [C]	N	Y	Y	N	N	Indexed<br>database search	N	N	Y	Miura*et al.*, 2005[7], [a];<br>Yamada *et al.*, 2006[8], [b];<br>Mann *et al.*, 2009[9], [b]
in silico PCR	N	N	Y	N	Y	Y	Indexed<br>database search	N	N	N	http://genome.ucsc.edu<br>/cgi-bin/hgPcr[b]
e-PCR	N	N	Y	N	N	Y	Indexed<br>database search	N	N	N	Schuler, 1997[10], [a];<br>Rotmistrovsky *et al.*, 2004[11], [b]
PCRTiler	Y [S]	N	Y	N	Y	Y	BLASTn	Y [C]	N	Y	Gervais *et al.*, 2010[12], [a]
ThermoAlign	Y [C]	N	Y	Y	Y	N	BLASTn;<br>thermoalignment	Y [C]	Y [S]	Y	https://github.com/drmaize/ThermoAlign[b]

[1]Y: yes; N: no; [C]Custom approach; [S]Relies on a secondary tool.

[2]NN model: nearest neighbor thermodynamic model.

[3]GENOMEMASKER, MFEprimer-2.0, PRIMEGENSw3, SDSS and e-PCR initiate the search specificity using a subsequence.

[4]For citations see the bibliography for the paper. Citations are highlighted as either an (a) original publication or a (b) software resource for the method or tool.

Table S2. Effects of the amplicon size range parameter on the minimum tiling path primer design for the 24 kb target region described in the main text.

	Amplicon size range (kb)						
	0.1-0.5	0.1-1.0	0.1-5.0	5-10	10-15	15-20	20-24
Number of possible primer pairs	1,463	3,505	400	348	81	0	0
Minimum amplicon length (bp)	100	100	100	5,000	10,043	0	0
Maximum amplicon length (bp)	496	990	5,000	9,603	14,839	0	0
Mean amplicon length (bp)	280	534	2,076	6,237	13,047	0	0
Median amplicon length (bp)	301	587	1,435	6,323	12,853	0	0
Cumulative amplicon coverage (bp)	2,699	4,530	8,566	14,840	14,840	0	0
Percent coverage	$\approx$11%	$\approx$19%	$\approx$36%	$\approx$62%	$\approx$62%	0	0
Number of subnetworks	5	3	1	1	1	0	0
Total number of primer pairs	9	9	4	3	1	0	0
[1]Percent coverage (A/T-end filter off)	$\approx$13.8%	$\approx$20%	$\approx$37%	$\approx$62%	$\approx$89%	$\approx$82%	0

[1]Coverage based on primer pairs designed when excluding the A/T-end filter as described in the results section on UOD.

Supplementary Figures

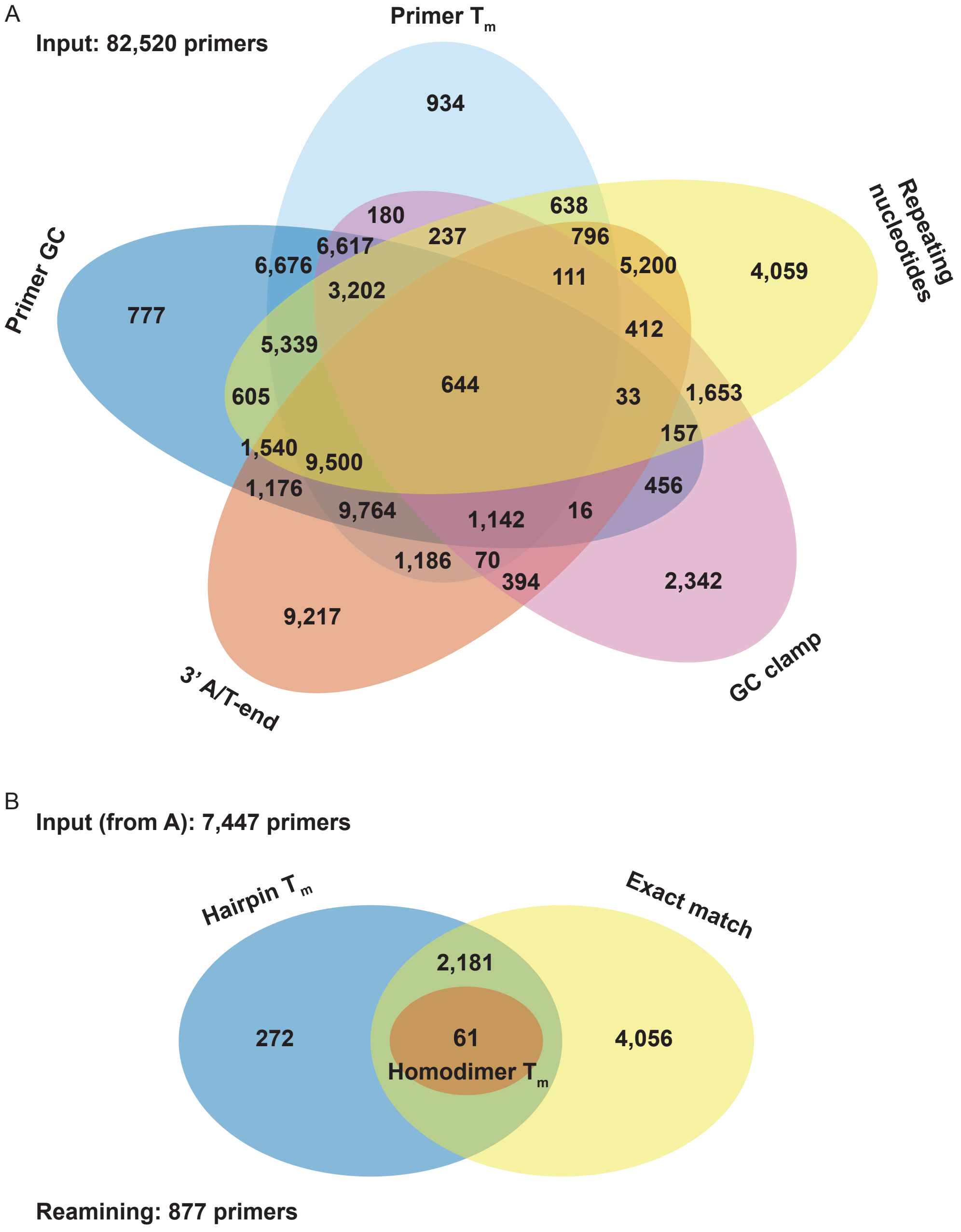

Figure S1. Effects of UOD filters on all 82,520 primers at monomoprhic sites across the 24 kb region described in the main text. The number of primers filtered are indicated for the parameters classified as **(A)** primer features and **(B)** primer interactions.

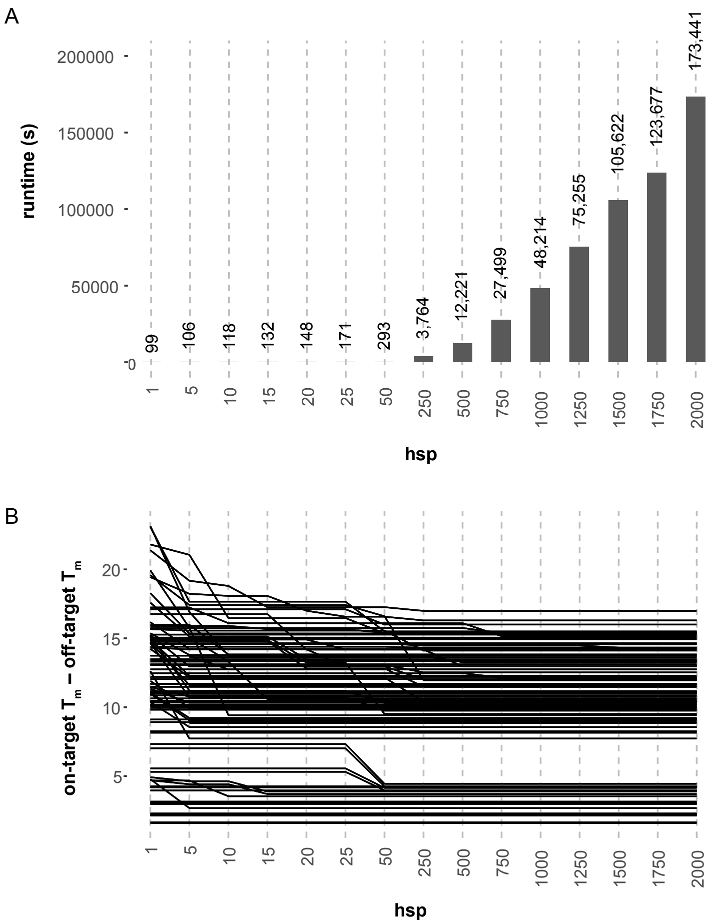

Figure S2. Relationship between ThermoAlign search speed and the search space required for a sufficient search to identify primers predicted to produce specific, on-target PCR products (i.e. on-target $T_m > 10°C$ of all off-target T_m values). Results are based on primers designed to the 24 kb locus described in the main text. **(A)** Wall time seconds for running ThermoAlign using different BLASTn hsp values for PSE. **(B)** Lines in the plot show the difference between on-target and off-target T_m (computed from thermoalignments) of each primer across different BLASTn hsp values used for PSE module. The search space is expanded by increasing the BLATSn hsp value. A single primer may have multiple off-target matches, but each primer is represented only by the off-target match with the minimum difference in T_m. Only primers that resulted in a change at different hsp values were plotted. Note: because hsp alignments with the same percent identify to the query sequence may be extended into thermoalignments that are not of the same percent identity, it is not guaranteed that the minimally distant off-target site will be identified when the hsp value is low. For this reason, some primers show a decreasing difference in T_m as the hsp settings is increased. Once there is no change, the minimally distant off-target site within the genome has been identified.

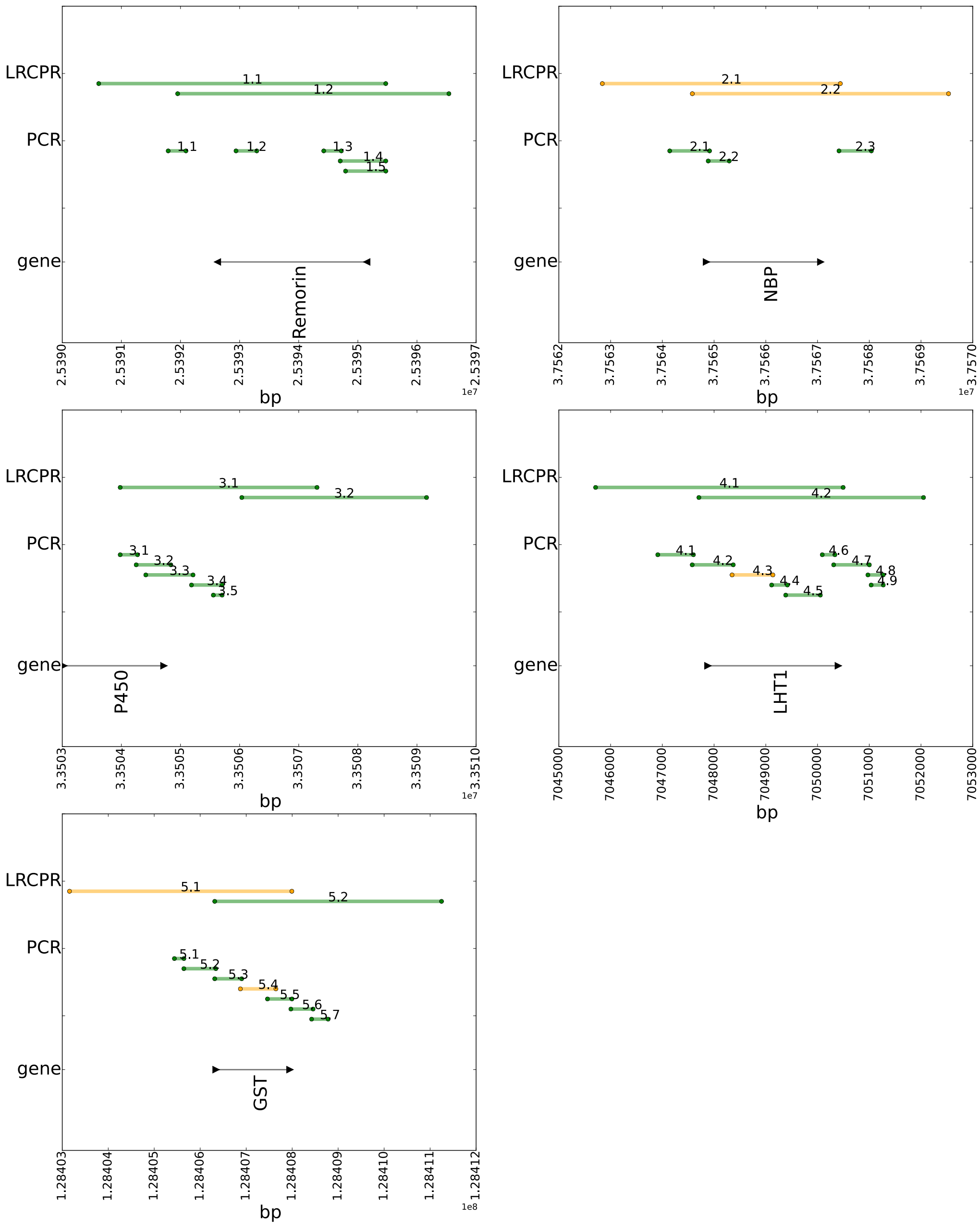

Figure S3. Minimum tiling paths of standard PCR and long range PCR primers tested in this study. Green lines indicate amplicons from primer pairs that worked without any reaction optimization. Orange lines indicate amplicons from primers that worked only with the addition of betaine. The gene track indicates genes to which the primer pairs were designed (Remorin: GRMZM2G107774; NPB: GRMZM2G022627; p450: GRMZM2G031364; LHT1: GRMZM2G127328; GST: GRMZM2G416632).

Supplementary methods and results

Region Specific Enrichment on maize

A Region Specific Enrichment (RSE) technology used to resequence a 150 kb section of the human genome[13] was tested on maize. Region specific enrichment involves hybridization of primers followed by single-strand synthesis with a mixture of standard and biotinylated nucleotides. The synthesized fragments, while still bound to the copied DNA, are pulled down with streptavidin-coated magnetic beads. Through this approach, the targeted DNA fragment is captured and enriched, uncoupled from the single-strand synthesis fragments, amplified using whole genome amplification and sequenced. Generation Biotech (Lawrenceville, NJ) was contracted to perform RSE on eight regions of the maize genome (Table S3). A pilot experiment was performed using six samples of maize, including the same B73 sample used for the reference genome[14]. Generation Biotech provided paired-end 100-bp sequence data produced on an Illumina HiSeq 2000 (reads are deposited at the NCBI sequence read archive; SRA ID: SRP048756, BioProject ID: PRJNA263415). The reads for each sample (here, we present results for the B73 sample) were quality trimmed using FASTX toolkit[15], mapped to the B73 reference genome (AGP v2) with bwa-mem[16] and summarized in terms of on-target versus off-target coverage using ”-hist” of bedtools[17] (Figure S4). Region specific enrichment showed effectively no enrichment of the target regions.

Table S3. Eight genomic loci in maize B73 genome, selected for targeted enrichment

Target site #	Target locus name	Chr.	Start pos. (B73_v3)	Stop pos. (B73_v3)	Length(bp)
1	qNLB_1_25376615_22184	1	25,376,615	25,398,798	22,184
2	qNLB_1_187278617_197947	1	187,278,617	187,476,563	197,947
3	qSLB_2_37556845_13001	2	37,556,845	37,569,845	13,001
4	qSLB_3_33490673_24001	3	33,490,673	33,514,673	24,001
5	qSLB_3_219917184_72001	3	219,917,184	219,989,184	72,001
6	qSLB_6_7002788_135001	6	7,002,788	7,137,788	135,001
7	qMDR_7_128386997_50394	7	128,386,997	128,437,390	50,394
8	qSLB_9_16206364_303882	9	16,206,364	16,510,245	303,882

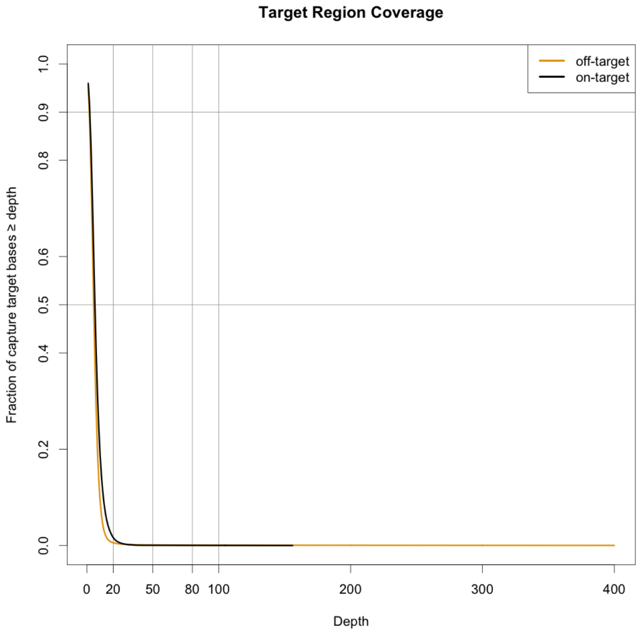

Figure S4. Depth of coverage for on-target versus off-target regions of the B73 reference genome from Illumina sequencing of RSE on B73 gDNA.

Follow-up experiments were performed to examine the potential cause for the lack of enrichment. For this, three on-target and three off-target qPCR assays (Applied Biosystems™ TaqMan®; Table S4) were used to more rapidly assess enrichment as the ratio between qPCR estimates of on-target and off-target DNA amounts. The RSE products were provided by Generation Biotech. Quantitative PCR was performed at the University of Delaware using TaqMan Gene Expression Master Mix and reagents (Applied Biosystems; Cat: #4369016) following the manufactures recommendations. Each assay was prepared in triplicate and read with an Applied Biosystems 7900HT Fast Real-Time PCR System.

When the primers for RSE were excluded from the reaction, gDNA was captured with approximately equal amounts of on-target and off-target DNA (Table S5). Estimates of the amount of enrichment by qPCR were not significantly different ($\alpha = 0.05$) for RSE with and without primers used for capture. When the same test was performed without primers but also excluding polymerase, no gDNA was captured (data not shown). Together, this suggested that our samples of maize gDNA led to non-specific single-strand synthesis products (required for capture via incorporation of biotinylated dNTPs), which in turn led to non-specific capture and failure to enrich the target regions.

Table S4. Target and off-target qPCR assays used to quantify enrichment ratios

Target / off-target	TaqMan assay name	Target gene accession #	Chr.	Target locus name
	Zm03998176_s1	NP_001148434	1	qNLB_1_187278617_197947
Target	Zm04041148_s1	NP_001140650	1	qNLB_1_187278617_197947
	Zm04077775_s1	NP_001136630	9	qSLB_9_16206364_303882
	Zm04080181_s1	NP_001147508	8	-
Off-target	Zm04040029_s1	NM_001157035	4	-
	Zm04084821_s1	NP_001150767	5	-

Table S5. Enrichment ratios of RSE products

B73 gDNA	Enrichment ratio ± SE (target/non-target DNA)
RSE with primers	4.13 ± 0.53
RSE without primers	2.44 ± 0.47

Selective whole genome amplification on maize

Selective whole genome amplification (SWGA) is a technique based on multiple displacement amplification (MDA) using primers prevalent in a target genome and rare in the background genome(s) in order to selectively amplify the target genome[18]. Using phi29 polymerase for MDA, this approach resulted in $>10^5$-fold amplification of the targeted *Borrelia burgdorferi* genome (as compared to <6.7 -fold background amplification) from a 1:2000 mixture of the *B. burgdorferi* and *Escherichia coli* genomes[18]. The efficacy of phi29-based MDA for targeted enrichment on maize (DNA from B73) was tested, considering the targeted region as the "target genome" and the non-targeted space as the "background genome." Forward and reverse primers flanking the target of two qPCR assays (Zm03998176_s1 and Zm04041148_s1 in Table S4) were designed for MDA. Off-target T_m for primers in the target region were calculated based on their BLASTn local alignments (not thermoalignments, as these tests were performed prior to the development of ThermoAlign) with the background genome, and primers with the maximum difference in target vs off-target T_m were used. qPCR based quantification of the amount of DNA corresponding to the target and non-target region indicated that the SWGA technology resulted in little to no enrichment of the target regions (Table S6). We note our study was not an attempt to validate the findings of Leichty et al.[18]. We consider our failure to achieve enrichment to be specific to our tests, which used a more complex genome and applied SWGA in a different manner than described by Leichty et al.[18]. We also tested this using primers designed prior to our development of ThermoAlign. Nevertheless, a difficulty of this approach with repetitive genomes like maize is identifying primers flanking the target region that will bind specifically at 30 °C, which is required for phi29 amplification.

Selective whole genome amplification was performed using phi29 DNA polymerase (New England Biolabs; Cat: #M0269L). Prior to assembling the reactions, template DNA was heated for 2 min at 90 °C and immediately placed on ice. Reactions (prepared on ice) with a final concentration of 1X phi29 reaction buffer, 0.2 mg/mL BSA, 1 mM dNTPs, 0.125, 0.250, or 0.500 μM forward and reverse primer set (total) and 30 U phi29 were combined with 50 ng of gDNA and brought to a volume of

50 µL with molecular-grade water. Amplification was carried out on an Eppendorf Mastercycler pro S with the following conditions: 5 min at 39 °C; 10 min at 38 °C; 15 min at 37 °C; 20 min at 36 °C; 30 min at 35 °C; 960 min at 34 °C; 15 min at 65 °C; and a hold at 4 °C. PCR products were assayed by electrophoresis in a 1% TBE gel and imaged using a FluorChem HD2 with AlphaView SA v3.4.0, which showed amplification had occurred (not shown).

Amplified products were cleaned with SPRIselect and qPCR was performed using TaqMan Gene Expression Master Mix and reagents (Applied Biosystems; Cat: #4369016) following the manufactures recommendations. Each assay was prepared in triplicate and read with an Applied Biosystems 7900HT Fast Real-Time PCR System.

Table S6. qPCR analysis of SWGA products

qPCR assay that the primers flanked	PCR primer concentration	Enrichment ratio ± SE (target/non-target DNA)
	500 nM	1.54 ± 0.23
**Zm03998176_s1**	250 nM	1.96 ± 0.12
	125 nM	1.71 ± 0.17
	500 nM	0.98 ± 0.05
**Zm04041148_s1**	250 nM	1.03 ± 0.06
	125 nM	1.02 ± 0.10
**No amplification (gDNA)**	N/A	1.23 ± 0.09

References

1. Untergasser, A. *et al.* Primer3-new capabilities and interfaces. *Nucleic Acids Res.* **40**, e115 (2012).
2. Andreson Eric Reppo, Lauris Kaplinski and Maido Remm, R. Software Open Access GENOMEMASKER package for designing unique genomic PCR primers. *BMC Bioinformatics* **7**, 172 (2006).
3. Qu, W. *et al.* MFEprimer-2.0: A fast thermodynamics-based program for checking PCR primer specificity. *Nucleic Acids Res.* **40**, W205–8 (2012).
4. Srivastava, G. P., Hanumappa, M., Kushwaha, G., Nguyen, H. T. & Xu, D. Homolog-specific PCR primer design for profiling splice variants. *Nucleic Acids Res.* **39**, e69 (2011).
5. Kushwaha, G., Srivastava, G. P. & Xu, D. PRIMEGENSw3: A web-based tool for high-throughput primer and probe design. *Methods Mol. Biol.* **1275**, 181–199 (2015).
6. Ye, J. *et al.* Primer-BLAST: A tool to design target-specific primers for polymerase chain reaction. *BMC Bioinformatics* **13**, 134 (2012).
7. Miura, F., Uematsu, C., Sakaki, Y. & Ito, T. A novel strategy to design highly specific PCR primers based on the stability and uniqueness of 3'-end subsequences. *Bioinformatics* **21**, 4363–4370 (2005).
8. Yamada, T., Soma, H. & Morishita, S. PrimerStation: A highly specific multiplex genomic PCR primer design server for the human genome. *Nucleic Acids Res.* **34**, W665–W669 (2006).
9. Mann, T., Humbert, R., Dorschner, M., Stamatoyannopoulos, J. & Noble, W. S. A thermodynamic approach to PCR primer design. *Nucleic Acids Res.* **37**, e95 (2009).
10. Schuler, G. D. Sequence mapping by electronic PCR. *Genome Res.* **7**, 541–550 (1997).
11. Rotmistrovsky, K., Jang, W. & Schuler, G. D. A web server for performing electronic PCR. *Nucleic Acids Res.* **32**, W108–12 (2004).
12. Gervais, A. L., Marques, M. & Gaudreau, L. PCRTiler: Automated design of tiled and specific PCR primer pairs. *Nucleic Acids Res.* **38**, W308–12 (2010).
13. Dapprich, J., Ferriola, D., Magira, E. E., Kunkel, M. & Monos, D. SNP-specific extraction of haplotype-resolved targeted genomic regions. *Nucleic Acids Res.* **36**, e94 (2008).
14. Schnable, P. S. *et al.* The B73 Maize Genome: Complexity, Diversity, and Dynamics. *Science (80-.).* **326**, 1112–1115 (2009).
15. Gordon, A. & Hannon, G. J. Fastx-toolkit. *FASTQ/A short-reads preprocessing tools (unpublished) http://hannonlab. cshl. edu/fastx_toolkit* (2010).
16. Li, H. Aligning sequence reads, clone sequences and assembly contigs with BWA-MEM (2013). 1303.3997.
17. Quinlan, A. R. & Hall, I. M. BEDTools: a flexible suite of utilities for comparing genomic features. *Bioinformatics* **26**, 841–842 (2010).
18. Leichty, A. R. & Brisson, D. Selective whole genome amplification for resequencing target microbial species from complex natural samples. *Genetics* **198**, 473–481 (2014).
